# Synthesis and Characterization of Zirconium Nitride Nanopowders by Internal Gelation and Carbothermic Nitridation

**DOI:** 10.1038/s41598-019-55450-x

**Published:** 2019-12-16

**Authors:** Shijiao Zhao, Jingtao Ma, Rui Xu, Xuping Lin, Xing Cheng, Shaochang Hao, Xingyu Zhao, Changsheng Deng, Bing Liu

**Affiliations:** 1 State Key Laboratory of New Ceramics and Fine Processing, Tsinghua University, 100084 Beijing, China; 2 Collaborative Innovation Center of Advanced Nuclear Energy Technology, Tsinghua University, 100084 Beijing, China; 3 Institute of Nuclear and New Energy Technology, Tsinghua University, Beijing, 100084 China

**Keywords:** Materials science, Synthesis and processing

## Abstract

Zirconium compounds has been widely attention over the last decades due to its excellent physical and chemical properties. Zirconium nitride nanopowders were synthesized via a simple direct carbothermic nitridation process of internal gel derived zirconia in the presence of nano-sized carbon black. The effects of reaction temperature, dwell time and molar ratio of carbon black to Zr (C/Zr) on the phase composition, grain size and crystal parameters of products were studied. Based upon the analysis of crystallite phase evolution and microstructure characterization, it was found that zirconium oxynitride is intermediate product and then O atoms in oxynitride were extracted by oxygen getter, carbon black. Anion sites were directly replaced by N atoms to form rock-salt type nitride in carbothermic nitridation process.

## Introduction

Transition metal nitrides are of great interest in various industry applications^1,2^ including packaging materials for semiconductors^3,4^, coatings for high-speed alloy cutting^5^, and high-temperature structural ceramics for nuclear materials^6^, etc., owing to their excellent physical, chemical, and mechanical properties^7–9^. Among them, zirconium nitride exhibits high hardness (~15 GPa)^10^, high melting point (2980 ± 50 °C), good thermal conductivity (45–50 W/mK)^11^, low electrical resistivity^12^, good abrasive resistance^13^ and good corrosion resistance. It has attracted a wide range of attention and has found many applications, such as coatings for thermal barrier layers and tooling setups for materials processing^14,15^, refractory materials^16^, diffusion barriers^17^, and Josephson junction in electronics^18^. Particularly, based on its low neutron capture cross-section and good chemical compatibility with actinides, zirconium nitride is an important material used as ceramic matrix of inert matrix fuel (IMF) to transmute long-lived actinides and as advanced fuel particle coatings^19–21^. Moreover, it is a surrogate for uranium nitride in order to optimize the process parameters for nitride fuel fabrication, which is being considered for application in space power reactors^22^ and advanced accident-tolerant fuels for nuclear reactors^23,24^.

The synthesis of zirconium nitride powders mainly includes direct nitridation of Zr metal with nitrogen^25,26^, high energy reactive ball milling (RBM)^27^, microwave plasma method^28^, benzene-thermal method^29^, aluminum reduction nitridation^30^, magnesium thermal reduction^31^, carbothermic reduction nitridation (CRN)^32^, and direct carbothermic nitridation (CN) of zirconia (ZrO_2_)^33^ and zircon^34^ etc. CRN and CN processes are appropriate routes for various sizes and morphologies like particles^35–37^, fibers^38^, microspheres^21^, films^39^ and bulk materials^20^, and have a great possibility of large-scale production of zirconium nitride and other transition metal nitrides. Because of the formation of solid solution in the ZrN-ZrC-‘ZrO’ system^40,41^, it should be noted that the final nitrided products in CRN or CN are in general represented by the formula Zr(N,C,O). CRN process needs two-step heat treatments by which zirconium carbide (ZrC) was first produced as an intermediate before conversion to nitride. However, CN process is direct nitridation of ZrO_2_ in the presence of carbon which needs only one heat treatment. The latter therefore could be more energy-efficient and time-saving for preparation of zirconium nitride powders.

The direct carbothermic nitridation of ZrO_2_ has been investigated by nitrogen absorption measurement^41^ and deconvolution of reaction progression using a novel thermogravimetric analysis (TGA) technique^42^. Even though the synthesis of Zr(N,C,O) by CN process and sol-gel was studied^33,41^, to the best of our knowledge, the particle size of prepared Zr(N,C,O) was in micrometer range, and very few studies analyzed the factors for the process of nitridation. Furthermore, the role of carbon in the CN process is still not very clear.

In this work, zirconium nitride nanopowders were synthesized by the CN process with internal gel derived precursor. Internal gelation process could offer homogeneous dispersion of the reaction moieties^43,44^ and could reduce dwell temperature and time of CN process^45^. The effects of nitridation temperature from 1300 °C to 1500 °C, dwell time from 2 h to 5 h, and the molar ratio of carbon black to Zr (C/Zr) from 0 to 3 on the powder characteristics were systematically studied. Microstructural analyses of nitrided products were conducted. Combined with phase evolution and microstructure evolution, the role of carbon in the CN process was discussed.

## Results and Discussion

### Effects of reaction temperature and dwell time

**Figure 1:**
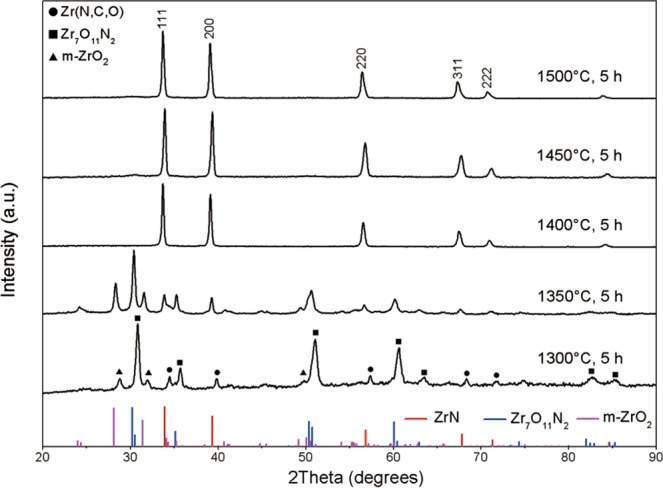
XRD patterns of samples treated at different temperatures for 5 h with C/Zr = 2.

The X-ray diffraction (XRD) patterns of samples treated at different nitridation temperature with C/Zr molar ratio of 2 are shown in Fig. 1. The molar ratio of C/Zr = 2 is the theoretical value for CN reaction (Eq. (1)).

1 $$2{{\rm{ZrO}}}_{2}({\rm{s}})+{{\rm{N}}}_{2}({\rm{g}})+4{\rm{C}}({\rm{s}})\to 2{\rm{ZrN}}({\rm{s}})+4{\rm{CO}}({\rm{g}})$$

At 1300 °C for 5 h, there were three phases i.e., zirconium oxynitride (Zr_7_O_11_N_2_) (JCPDS file no 00-048-1637), monoclinic zirconia (m-ZrO_2_) (JCPDS file no 00-037-1484), and zirconium nitride with rock-salt type cubic structure (JCPDS file no 00-035-0753), which also contained C and O as solid solution atoms in the cubic structure as stated above, and it was represented by Zr(N,C,O) there. The presence of Zr_7_O_11_N_2_ was in line with the results of previous studies^41,46^. At 1350 °C, though relative content of nitride phase increased compared with sample in 1300 °C, oxynitride phase still the main phase in product. Those temperatures may be insufficient for completion of nitride formation in 5 h. When the temperature was increased to 1400 °C, sample with Zr(N,C,O) phase was obtained as shown in XRD pattern. Quantitative analysis of this sample showed that light elements C, N, and O contents were 13.23 wt%, 7.32 wt% and 2.77 wt%, respectively. It could be pointed out that free carbon resided in the sample. Thus a relative high C content was determined. The free carbon is amorphous. Its diffraction intensity is much lower than that of crystalline nitride phase so that no diffraction peaks of free carbon could be observed in XRD pattern. Microscopy will provide its existence in the latter section. Further increase in temperature to 1450 °C and 1500 °C, the products also were both zirconium nitride phase, Zr(N,C,O). Besides, from these XRD patterns, the grain size of the products at 1400 °C, 1450 °C, and 1500 °C was calculated by Debye-Scherrer formula as shown in Eq. (2).

2 $${\rm{D}}={\rm{K}}\gamma /{\rm{Bcos}}{\rm{\theta }}$$

In Eq. (2), B represents the half-width of the highest diffraction peak of zirconium nitride corresponding to (111) plane and the instrumental correction factor is −0.104. And K, γ and θ represent Scherrer constant (0.89), wavelength of radiation (1.54056 Å) and diffraction angle, respectively. The calculation results are presented in Table 1. The results suggest that all of the nitride powders were nanocrystalline.

**Table 1:** Grain size of products of zirconium nitride phase.

Temperature (°C)	Dwell time (h)	Grain size (nm)
1400	5	75
1450	5	50
1500	5	54

The error is about 1 nm.

**Figure 2:**
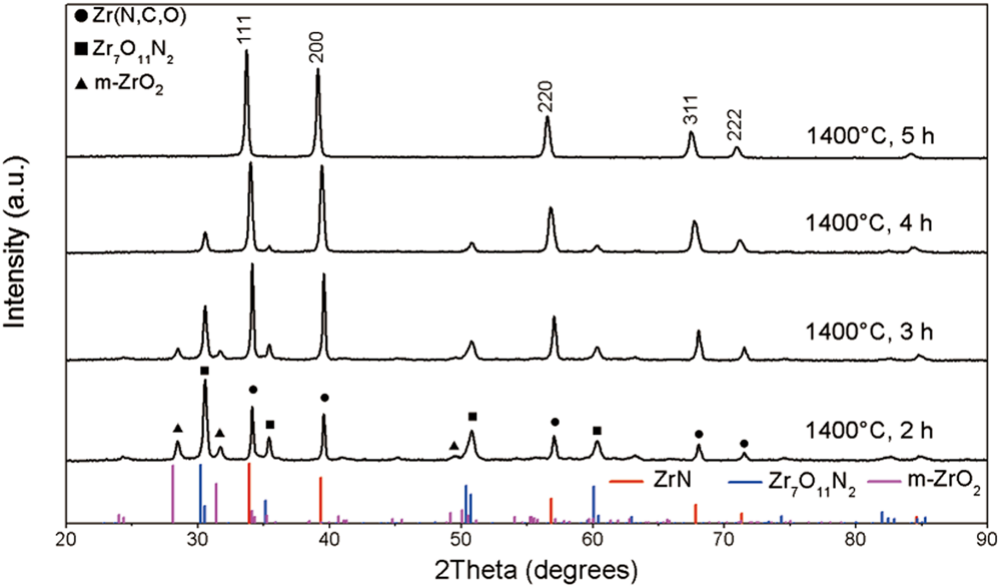
XRD patterns of samples treated at 1400 °C for different dwell time with C/Zr = 2.

Figure 2 shows the effect of dwell time. At 1400 °C, with the time for heat treatment increased from 2 h to 5 h, the relative peak intensity for Zr_7_O_11_N_2_ (122) at 2theta = 30.2° gradually decreased, and that for Zr(N,C,O) (111) at 2theta = 33.9° increased. It corresponds to the increase in relative content of rock-salt phase with the increase in dwell time. Oxynitride, Zr_7_O_11_N_2_, was produced in the early nitridation process, then decreased with further nitridation. Besides, no diffraction peaks associated with zirconium carbide were found. It is naturally deduced that oxynitride compound is intermediate product in CN process and it was gradually converted to nitride phase till completed nitridation.

### Effects of molar ratio of C/Zr

**Figure 3:**
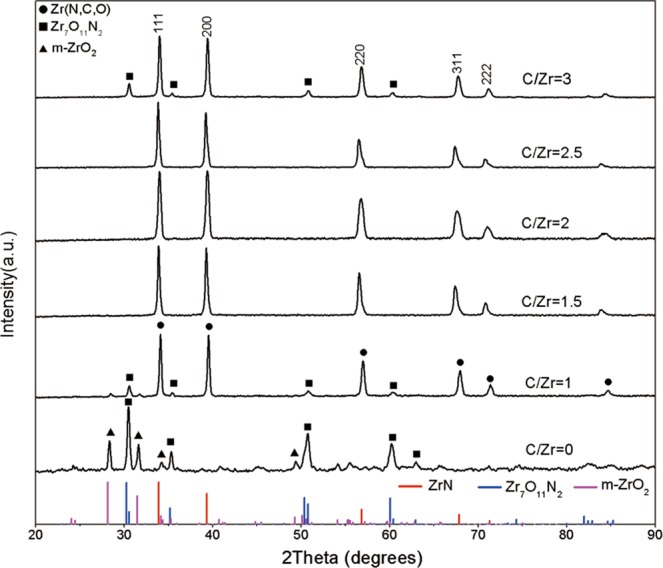
XRD patterns of samples treated at 1400 °C for 5 h with different C/Zr (0–3).

Figure 3 shows the effect of the molar ration of C/Zr on the phase composition of products treated at 1400 °C for 5 h. Different molar ratios of C/Zr = 0, 1, 1.5, 2, 2.5 and 3 were selected. As shown in Fig. 3, when C/Zr = 0, i.e., no carbon black was added in the reactant, the phase composition was Zr_7_O_11_N_2_ and m-ZrO_2_. Zirconia can be partially nitrided in nitrogen and zirconium oxynitride formed as shown in Eq. (3)^47^,

3 $${{\rm{ZrO}}}_{2}+(2{\rm{x}}/3){{\rm{N}}}_{2}\rightleftharpoons {{\rm{ZrO}}}_{2-2{\rm{x}}}{{\rm{N}}}_{4{\rm{x}}/3}+{{\rm{xO}}}_{2}$$

If nitride was to be produced in the absence of carbon, the reaction temperature needed to be up to 2000 °C or even higher^48^. In our study, nitride appeared in the product with C/Zr = 1 at 1400 °C. It shows the necessity of the presence of carbon black for preparation of nitride at such reaction temperature. However, there was still significant amount of oxynitride. When the molar ratio of C/Zr was increased to 1.5, 2 and 2.5, the products were Zr(N,C,O). Further increasing C/Zr value to 3, the diffraction peaks of Zr_7_O_11_N_2_ appeared again. This result indicates that whether lower or higher value was not conducive to CN process. Specifically, addition of carbon into reactant is necessary for formation of nitride. Increasing the amount of carbon improves the nitridation level. But excess carbon is harmful to complete carbothermic nitridation, which decreased the contact area of zirconia and gas medium, resulting in a much longer pathway for nitrogen diffusion.

According to above XRD results of samples in different CN process conditions, lattice parameters of nitride phase in samples were determined as shown in Table 2. As we known, in ZrN-ZrC-‘ZrO’ system, lattice parameters of ZrN, ZrC and ‘ZrO’ are 4.578 Å (JCPDS file no 00-035-0753), 4.693 Å (JCPDS file no 00-035-0784) and 4.626 Å (JCPDS file no 00-051-1149), respectively. The range of lattice parameters was 4.524–4.607 Å. This range further shows that nitrides in CN process were solid solution with C and O atoms, i.e. Zr(N,C,O). The columns with bold words in Table 2 are samples with Zr(N,C,O) phase according to XRD patterns. Among them, the lattice parameter of sample with C/Zr molar ratio 1.5 heated at 1400 °C for 5 h (4.575 Å) is the closest to the standard ZrN, and has the lowest solid solution content of C and O.

**Table 2:** Lattice parameters of Zr(N,C,O) in different samples.

Temperature (°C)	Dwell time (h)	C/Zr molar ratio	Lattice parameter (Å)
1300	5	2	4.524
1350	5	2	4.594
1400	2	2	4.559
1400	3	2	4.564
1400	4	2	4.575
1400	5	1	4.584
***1400***	***5***	***1.5***	***4.575***
***1400***	***5***	***2***	***4.600***
***1400***	***5***	***2.5***	***4.560***
1400	5	3	4.575
***1450***	***5***	***2***	***4.583***
***1500***	***5***	***2***	***4.607***

(The columns with bold words are products of zirconium nitride phase according to XRD patterns).

The error is about 1 × 10^−3^ Å.

### Microstructure of under-reacted product

**Figure 4:**
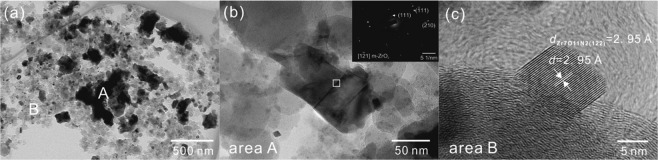
Microscopic images of under-reacted product obtained at 1350 °C for 5 h with C/Zr = 2. (**a**) Overview image; (**b**) morphology of angular-shaped particle and associated SAED pattern, corresponding to area A in (**a**); (**c**) HRTEM image of nanocrystalline in this product, corresponding to area B in (**a**).

**Figure 5:**
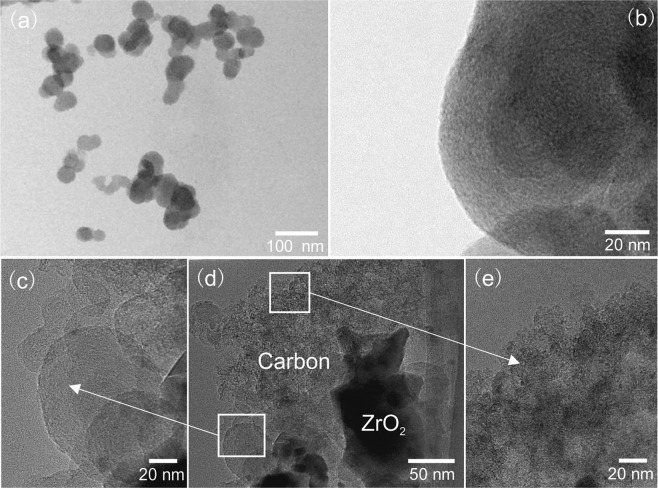
Microscopic images of carbon black particles. (**a**) Overview and (**b**) HRTEM image of original carbon black particles; (**c**~**e**) HRTEM images of carbon black particles in under-reacted product obtained at 1350 °C for 5 h.

As shown in XRD patterns in Fig. 1, the sample treated at 1350 °C for 5 h was under-reacted which consisted of Zr_7_O_11_N_2_, m-ZrO_2_ and Zr(N,C,O). Figure 4 shows the morphology of the sample treated at 1350 °C for 5 h. Figure 4(a) shows the angular-shaped particles (~200 nm), corresponding to area A, and nanocrystallites (~10 nm) mixed with residual amorphous carbon, corresponding to area B. Figure 4(b) is an enlarged image of an angular-shaped particle in area A. The selected area electron diffraction (SAED) pattern inserted in Fig. 4(b) indicates that the selected area was orientated along the 12I zone axis of m-ZrO_2_. It shows the twin crystals which are classically seen in *m*-ZrO_2_^49^. In Fig. 4(c), the high-resolution transmission electron microscopy (HRTEM) image of nanocrystallites demonstrated that it is Zr_7_O_11_N_2_. The interplanar distances *d* = 2.95 Å corresponds to *d*_Zr7O11N2(122)_ = 2.95 Å. In addition, carbon black as a crucial reactant in CN process, had obvious evolution in its morphology in CN process. Figure 5(a) demonstrated that the original carbon black particles were spherical with uniform particle size around 50 nm. A high revolution image in Fig. 5(b) shows a smooth surface and amorphous feature of the original carbon particles. After heat treatment, free carbon resided in products as elements analysis indicated in above section. Figure 5(d) shows residual carbon in the sample treated at 1350 °C for 5 h. Two features were found, marked in square regions in Fig. 5(d), which were different from the original carbon particles. One is carbon spheroids with decreased thickness and jagged surface, as magnified in Fig. 5(c). The other co-existing feature is flocculent carbon, as shown in Fig. 5(e). They indicated the morphology evolution of carbon black in CN process. In specific, carbon black participated in CN reaction and was consumed gradually at elevated temperatures, which rendered that the carbon particles lose their sphericity, surface became unstable and further particles disintegrated and flocculent carbon formed.

### Microstructure of fully reacted product

**Figure 6:**
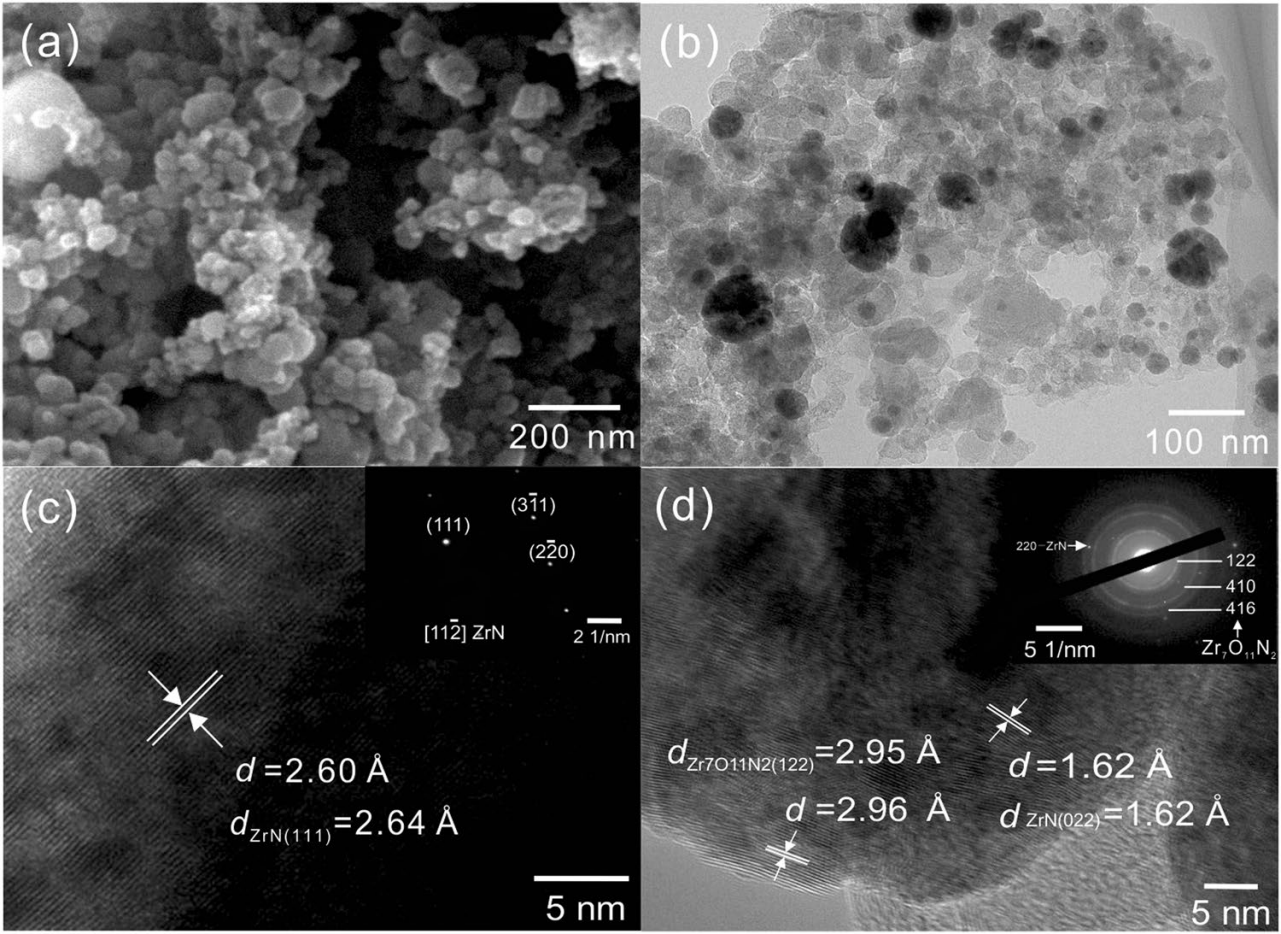
Microscopic images of fully reacted product obtained at 1400 °C for 5 h. (**a**) FE-SEM image; (**b**) TEM image; (**c**,**d**) HRTEM images and associated SAED patterns of product.

Preliminary scanning electron microscopy (SEM) observations found that fully reacted samples treated at 1400 °C, 1450 °C and 1500 °C for 5 h had similar morphology. In view of morphology similarity of the three powders, the microscopic images for the fully reacted product with C/Zr = 2, treated at 1400 °C for 5 h are presented in Fig. 6. As the SEM image shows in Fig. 6(a) that, the final Zr(N,C,O) powders were nanoparticles that tended to agglomerate. The particle size of the nanocrystalline as shows in Fig. 6(a) was in agreement with calculation results presented in Table 1. The overview TEM image shows in Fig. 6(b) that, the powders were composed of nanocrystallites and residual carbon. The morphology of carbon was similar to the carbon in under-reacted product. But crystalline zirconia particles with angular-shape disappeared.

HRTEM images of nanocrystallites are presented in Fig. 6(c,d). From Fig. 6(c), a distinct interplanar distance of *d* = 2.60 Å can be measured, almost identical to the *d*_ZrN(111)_ = 2.64 Å. Besides, the SAED pattern inserted in Fig. 6(c) clearly points out the presence of nitride with face-centered cubic structure, and this area of the particle was orientated along the 11$\bar{2}$ zone axis. Some particles had co-existence of nitride and oxynitride, as shown in Fig. 6(d). HRTEM image in Fig. 6(d) shows two obvious interplanar distances of *d*_1_ = 1.62 Å and *d*_2_ = 2.96 Å, which correspond to *d*_ZrN(220)_ = 1.62 Å and *d*_Zr7O11N2(122)_ = 2.95 Å, respectively. The SAED analysis inserted in Fig. 6(d) shows good consistency with the result obtained from HRTEM imaging analysis. The XRD pattern of the powder produced after heat treatment at 1400 °C in Fig. 1 only shows the diffraction peaks of nitride because of low relative content of Zr_7_O_11_N_2_ in the product. It is once again noted that attempts to find any crystalline characteristics that could be linked to zirconium carbide failed.

### The role of carbon in CN process

As has been shown in previous section, carbon plays a vital role in nitridation processes. Two nitridation method with carbon source are CRN process and CN process as mentioned. CRN is a two-step process, by which ZrO_2_ is firstly transformed to carbide through carbothermal reduction of ZrO_2_ in argon atmosphere following Eq. (4), then the obtained carbide is converted to nitride through nitridation by treatment in nitrogen-hydrogen mixing atmosphere following Eq. (5)^11^. Therefore, carbon not only acts as a reducing agent to take away O but also bonds with Zr atoms to form carbide as an intermediate product. The pathway of formation of nitride is anion sites substitution from C to N and take C away as HCN(g) simultaneously.

4 $${{\rm{ZrO}}}_{2}({\rm{s}})+3{\rm{C}}({\rm{s}})\to {\rm{ZrC}}({\rm{s}})+2{\rm{CO}}({\rm{g}})$$

5 $$2{\rm{ZrC}}({\rm{s}})+2{{\rm{N}}}_{2}({\rm{g}})+{{\rm{H}}}_{2}({\rm{g}})\to 2{\rm{ZrN}}({\rm{s}})+2{\rm{HCN}}({\rm{g}})$$

However, the role of carbon in CN process, i.e. direct nitridation in the presence of carbon as shown in Eq. (1), is yet to be clarified. Does it have the same role of carbon in CRN process? Based on our findings, the answer is negative and the evolution and the effect of carbon is discussed here.

First of all, the XRD patterns and TEM characterization both showed that no carbide was detected in the products after CN reaction. The existing forms of C in products are free carbon and solid solution atom in Zr(N,C,O). Secondly, rather the early appearance of oxynitride and its morphology in the products reveal that oxynitride is an intermediate in the CN process. This indicates that the formation of nitride in CN reactions did not go through the conversion pathway by which carbide was produced before nitride. The difference between CN and CRN is the intermediate. Thirdly, as oxynitride is intermediate product in CN process, N directly replace anion sites of O in the conversion from zirconia to oxynitride. Then this kind of substitution continues in the conversion from oxynitride to nitride. The conversion from zirconia to oxynitride could be realized when C/Zr = 0. But no nitride form in this case means addition of carbon black is prerequisite for the conversion from oxynitride to nitride.

The microstructure evolution of carbon black indicates that it is a participant in the CN reaction. It reveals that main role of carbon was an oxygen getter based on above three crucial points. The presence of carbon drives the direct replacement of O by N continuously till complete transformation to zirconium nitride was achieved. The carbon particles in nitrided products gradually reduced their size and their morphology evolved towards that appearing amorphous, flocculent and some with jagged surface. On the other hand, too excessive amount of residual carbon could reduce the contact area between nitrogen and zirconia, and hinder solid state diffusion, consequently is not conducive to the completion of CN reaction.

## Conclusions

Zirconium nitride nanopowders were prepared by carbothermic nitridation process of powder mixture of zirconium hydroxide gel with carbon black. The effects of temperature, dwell time and C/Zr molar ratio on carbothermic nitridation process were investigated. The relative content of nitride phase and the nitridation level increased with the increase of reaction temperature and dwell time. The molar ratio of C/Zr crucially decided the final composition of the powder products. The addition of carbon black is necessary for conversion from oxynitride to nitride. But excess value of C/Zr would impede solid state diffusion so that was harmful to further nitridation. Based on our research, zirconium oxynitride is intermediate product in CN process, and carbon mainly acted as an oxygen getter in this process. Those are the differences from CRN process. The CN process started with nucleation of oxynitride and substitution of its oxygen atoms by nitrogen completed the nitride formation under the action of carbon as an oxygen getter.

## Materials and Methods

### Experimental

ZrO(NO_3_)_2_·xH_2_O (99.5%, Aladdin, Shanghai, China) was chosen as the zirconium source and carbon black (TPX-1408, Cabot Chemical, Massachusetts, USA) was used as the carbon source. Urea (≥99.0%, Sinopharm Chemical Reagent, Beijing, China) was used as a chelating agent of zirconium. Hexamethylenetetramine (HMTA) (≥99.0%, Yongda Chemical Reagent, Tianjin, China) was used as a hydrolyzing agent.

Solution 1 was prepared by dissolving ZrO(NO_3_)_2_·xH_2_O in deionized water to give 1.6 mol/L ZrO^2+^. Solution 2 containing 3 mol/L HMTA and 2.625 mol/L urea in deionized water was also prepared. Then carbon black was added into 20 mL of Solution 2 and dispersed ultrasonically for 15 min. The molar ratio of HMTA to zirconium element (HMTA/Zr) was 1.3. Molar ratio of urea to zirconium (urea/Zr) was set at 1.14 according to our previous work^43^. The C-containing liquid was added dropwise into 28.7 mL of Solution 1 under stirring. The addition of carbon-containing liquid triggers the hydrolysis of ZrO(NO_3_)_2_ (Eq. (5)), which was catalyzed by OH^−^ ions produced by the decomposition of HMTA (Eq. (6)).

5 $${{\rm{ZrO}}}^{2+}+2{{\rm{H}}}_{2}{\rm{O}}\to {\rm{ZrO}}{({\rm{OH}})}_{2}+2{{\rm{H}}}^{+}$$

6 $${({{\rm{CH}}}_{2})}_{6}{{\rm{N}}}_{4}+10{{\rm{H}}}_{2}{\rm{O}}\to 4{{\rm{NH}}}_{4}^{+}+4{{\rm{OH}}}^{-}+6{{\rm{CH}}}_{2}{\rm{O}}$$

Subsequent condensation of ZrO(OH)_2_ forms three-dimensional Zr-O-Zr gel network. Carbon particles were incorporated and intimately mixed with the Zr-O-Zr gel network. The molar ratio of carbon black to zirconium (C/Zr) was set as 0~3, with an increment of 0.5. All the steps mentioned above were carried out at ambient temperature.

The gel was dried in air at 60 °C for 12 h and subsequently ground into powders in a mortar for 20 min. Then, the carbon-containing gel powders were treated at different temperatures for 2~5 h using an alumina tube furnace (SENTRO Technologies, USA). The heat treatment was carried out first under flowing argon (99.99%) to remove residual organics, then the atmosphere was switched to nitrogen (99.99%) when the temperature rose to 800 °C. Nitrogen was still used during cooling to prevent oxidization. The heating rate and cooling rate were both 10 °C/min, and the flowing rate of argon and nitrogen were 100 mL/min and 300 mL/min, respectively.

### Characterization

The crystalline phase identification was carried out by X-ray diffraction (XRD, D8 Advance, Bluker, Germany) with Cu Kα radiation (λ = 1.54056 Å) at 40 kV for the angle (2θ) ranging between 20° and 90° and with a scanning rate 2°/min. Samples were ground in an agate mortar for 20 min before XRD characterization. Determination of lattice parameter was using Jade 6 software based on XRD pattern. C content was measured by elemental analyzer (CS744, LECO, USA) and N and O were measured by elemental analyzer (ON736, LECO, USA). Field-emission scanning electron microscope (FE-SEM, Merlin, Zeiss, Germany) was used to characterize the morphology of final powder products. The microstructure and diffraction patterns of the powder products were analyzed using high-resolution transmission electron microscope (HRTEM, JEM-2100F, JEOL, Japan), equipped with selected area electron diffraction (SAED).
